# Epidemiology and prevalence of tobacco use in Tehran; a report from the recruitment phase of Tehran cohort study

**DOI:** 10.1186/s12889-023-15629-4

**Published:** 2023-04-21

**Authors:** Akbar Shafiee, Alireza Oraii, Arash Jalali, Farshid Alaeddini, Soheil Saadat, Farzad Masoudkabir, Masih Tajdini, Haleh Ashraf, Negar Omidi, Amirhossein Heidari, Alireza Sepehri Shamloo, Saeed Sadeghian, Mohamamdali Boroumand, Ali Vasheghani-Farahani, Abbasali Karimi, Oscar H. Franco

**Affiliations:** 1 Tehran Heart Center, Cardiovascular Diseases Research Institute, Tehran University of Medical Sciences, Tehran, Iran; 2 Department of Emergency Medicine, University of California, Irvine, CA USA; 3 Cardiac Primary Prevention Research Center, Cardiovascular Diseases Research Institute, Tehran University of Medical Sciences, Tehran, Iran; 4 Faculty of Medicine, Tehran Medical Sciences, Islamic Azad University, Tehran, Iran; 5 Department of Electrophysiology, Heart Center Leipzig at University of Leipzig, Leipzig, Germany; 6 Institute of Social and Preventive Medicine (ISPM), University of Bern, Bern, Switzerland

**Keywords:** Tobacco, Cigarette, Waterpipe, Pipe, Epidemiology, Tehran

## Abstract

**Introduction:**

Tobacco use is a major health concern worldwide, especially in low/middle-income countries. We aimed to assess the prevalence of cigarette smoking, waterpipe, and pipe use in Tehran, Iran.

**Methods:**

We used data from 8272 participants of the Tehran Cohort Study recruitment phase. Tobacco use was defined as a positive answer to using cigarettes, waterpipes, or pipes. Participants who did not report tobacco use during the interview but had a previous smoking history were categorized as former users. Age- and sex-weighted prevalence rates were calculated based on the national census data, and characteristics of current and former tobacco users were analyzed.

**Results:**

Age- and sex-weighted prevalence of current tobacco users, cigarette smokers, waterpipe, and pipe users in Tehran was 19.8%, 14.9%, 6.1%, and 0.5%, respectively. Current tobacco use was higher in younger individuals (35–45 years: 23.4% vs. ≥ 75 years: 10.4%, *P* < 0.001) and men compared to women (32.9% vs. 7.7% *P* < 0.001). The prevalence of tobacco use increased with more years of education (> 12 years: 19.3% vs. illiterate: 9.7%, *P* < 0.001), lower body mass index (< 20 kg/m^2^: 31.3% vs. ≥ 35 kg/m^2^: 13.8%, *P* < 0.001), higher physical activity (high: 23.0% vs. low: 16.4%, *P* < 0.001), opium (user: 66.6% vs. non-user: 16.5%, *P* < 0.001), and alcohol use (drinker: 57.5% vs. non-drinker: 15.4%, *P* < 0.001). Waterpipe users were younger (46.1 vs. 53.2 years) and had a narrower gender gap in prevalence than cigarette smokers (male/female ratio in waterpipe users: 2.39 vs. cigarette smokers: 5.47). Opium (OR = 5.557, *P* < 0.001) and alcohol consumption (OR = 4.737, *P* < 0.001) were strongly associated with tobacco use. Hypertension was negatively associated with tobacco use (OR = 0.774, *P* = 0.005).

**Conclusion:**

The concerning prevalence of tobacco use in Tehran and its large gender gap for cigarette and waterpipe use warrant tailored preventive policies.

**Supplementary Information:**

The online version contains supplementary material available at 10.1186/s12889-023-15629-4.

## Implications

Tobacco use is among the leading risk factors for non-communicable diseases. Despite various reports from Iran, few epidemiological studies have described the prevalence of tobacco smoking in Tehran. We investigated the epidemiology of current and former cigarette smoking, waterpipe, and pipe use in adult residents of Tehran who participated in the Tehran Cohort Study. Among current tobacco users, 1231 (77.0%) were cigarette smokers, 449 (28.1%) were waterpipe users, and 44 (2.8%) were pipe users. Tobacco use was widespread in younger participants, men, those with higher levels of education, lower BMI, higher physical activity, and opium and alcohol users. Waterpipe users were considerably younger and had a lower male/female ratio than cigarette smokers.

## Introduction

Tobacco use is among the leading risk factors for attributable disability-adjusted life-years and an important preventable cause of death from non-communicable diseases [1, 2]. Approximately 1.3 billion people worldwide are tobacco users, of whom over 80% reside in lower and middle-income countries [3, 4]. Cigarettes are the most commonly used tobacco product globally, and waterpipe (also known as hookah, narghile, or shisha) has become increasingly popular, especially among the young generations [5]. Evidence suggests that waterpipe use results in adverse health outcomes similar to or even greater than cigarette smoking [6–8].

Epidemiological studies have reported a global decline in smoking prevalence over the past decades [2, 9]. While high-income countries have had more pronounced reductions, the prevalence of tobacco use has only changed slightly in Iran [10, 11]. Iran is a middle-income country in the Middle East with high variability in tobacco use across its different geographical regions. Tehran, Iran's capital city, is one of the most heavily-populated multi-ethnic urban areas in the Middle East. Few epidemiological studies have previously investigated the prevalence of tobacco smoking in Tehran [12, 13]. However, these studies were limited by district-level sampling, small study participants, or the inclusion of specific age groups. Moreover, cigarette smoking was the only type of tobacco assessed in some of these studies. Hence, there is a paucity of data on the smoking prevalence of other tobacco products among adult residents of Tehran. Additionally, the prevalence of comorbid conditions such as coronary heart disease has not been evaluated among tobacco users in Tehran.

To better understand the status of tobacco use in Tehran and its variation within subgroups, this study aimed to investigate the epidemiology of current and former cigarette smoking, waterpipe, and pipe use in adult residents of Tehran who participated in the Tehran Cohort Study (TeCS).

## Methods

### Study design and setting

The present study used data from the recruitment phase of TeCS, a cohort of adult citizens of Tehran city, the capital of Iran. The study rationale and protocol have been published elsewhere [14]. In brief, a systematic random sampling method was implemented to recruit households that include adult citizens aged ≥ 35 years from all districts of Tehran. A total of 4215 households, including 8296 adults aged ≥ 35 years, participated in TeCS between May 2016 and February 2019. The deputy of research and committee of ethics of Tehran University of Medical Sciences approved the protocol of TeCS (IR.TUMS.MEDICINE.REC.1399.074). All participants signed a written informed consent to participate.

### Study participants and data collection

Data of 8296 individuals aged ≥ 35 years who participated in TeCS were screened for inclusion. Those lacking information about cigarette, waterpipe, or pipe use were excluded. Demographic data, level of education, preexisting comorbidities, metabolic risk factors, and physical activity status were obtained through in-person interviews using predesigned questionnaires. Participants were questioned regarding the current or former use of any type of tobacco product, including cigarettes, waterpipes, and pipes. Moreover, tobacco smoking behaviors, including frequency of usage and duration of smoking, were investigated. Anthropometric characteristics, including height, weight, and body mass index (BMI), were also assessed.

### Definitions

Current cigarette smoking was defined as the self-reported regular or occasional use of tobacco cigarettes at the time of the interview. Those who did not report smoking at the time of the interview but gave a history of smoking or had quit smoking were defined as former cigarette smokers. Pack-year was calculated as the number of cigarette packs smoked per day multiplied by the duration of smoking in years. Waterpipe and pipe use (either current or former) were defined as similar to cigarette smoking. Current tobacco use was defined as the current use of any tobacco products mentioned above. Former tobacco users were those with any previous use of cigarettes, waterpipes, or pipes in the absence of current tobacco use.

Preexisting comorbidities included hypertension, diabetes mellitus, dyslipidemia, chronic kidney disease, or chronic lung disease (chronic obstructive pulmonary disease or asthma). These data were obtained via the participant's self-report or the current use of any related medications. Coronary artery disease (CAD) was considered positive in those who provided any documented evidence of significant coronary artery lesion or ischemic heart disease based on coronary artery angiograms, computed tomography angiography, myocardial perfusion imaging, or previous history of coronary revascularization (percutaneous coronary intervention or coronary artery bypass graft surgery). Opium consumption was defined as any use of opium or its derivatives. Alcohol consumption was defined as drinking alcoholic beverages regularly or occasionally. Physical activity was categorized as low, intermediate, and high activity based on a self-explained Likert-scale questionnaire.

### Statistical analysis

Categorical variables were reported as numbers with percentages, and age as a continuous variable was presented as mean ± standard deviation (SD). The Chi-squared test or Fisher exact test was used to compare categorical variables between the groups. The variable age was compared between the 3 groups of tobacco usage by applying a one-way analysis of variance (ANOVA) test. Also, the variable age was compared between the two former and current smoking groups of various types of smoking using independent samples t-test. Due to the different age and sex distribution of our study population compared with the adult population of the Tehran, age- and sex-weighted prevalences were calculated for current tobacco users, cigarette smokers, waterpipe, and pipe users by utilizing data from Tehran's adult population aged ≥ 35 years in the 2016 national census. The proportions of current and former tobacco use, cigarette smoking, waterpipe, and pipe use were analyzed and compared between various ages, sex, BMI, preexisting comorbidities, behavioral risk factors, physical activity, and education levels to explore variations in the mentioned proportions in these subgroups. The adjusted association of the variables with current tobacco use was assessed by applying a logistic regression model and the associations were reported through odds ratio (OR) with a 95% confidence interval (CI). All statistical analyses were performed using IBM SPSS Statistics for Windows, v.25.0 (IBM Corp., Armonk, NY, USA). The geographical distribution of current and former cigarette/waterpipe users was depicted in the Tehran map using the first three digits of the postal code using shp2dta and spmap modules in STATA release 14.2 (College Station, TX: Stata Corp LP.).

## Results

Among the total 8296 individuals enrolled in TeCS, 24 participants lacked information about cigarette, waterpipe, or pipe use. Hence, data from 8272 participants were analyzed in the present study. The study population had a mean age of 53.8 ± 12.74 years, and 54.0% were women. The baseline characteristics of the total study population are shown in Supplementary Table 1.

### Tobacco use

**Fig. 1:**
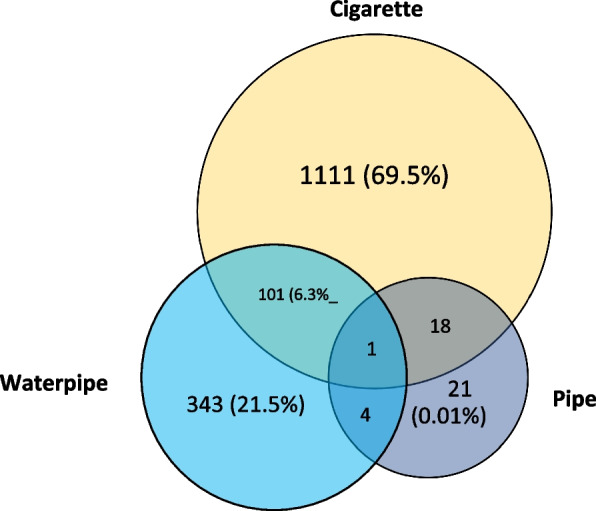
Detailed information about the number of current tobacco users in the Tehran Cohort study

A total of 1599 (19.3%) participants were current tobacco users in our study population, and the age- and sex-weighted prevalence of current tobacco use was 19.8% (95% CI: 18.0 – 21.6) in Tehran. Among current tobacco users, 1231 (77.0%) were cigarette smokers, 449 (28.1%) were waterpipe users, and 44 (2.8%) were pipe users. Further details on current tobacco users are depicted in Fig. 1. The prevalence of current and former tobacco users within baseline characteristic subgroups are shown in Table 1.

**Table 1:** Baseline characteristics of the study population according to tobacco use status

Characteristic	No tobacco use (*n* = 6339 [76.6%])	Former tobacco use (*n* = 334 [4.0%])	Current tobacco use (*n* = 1599 [19.3%])	*P*-value*
**Age, year**	53.9 ± 12.74	61.0 ± 13.22	51.7 ± 12.02	< 0.001
**Age category, year**				< 0.001
35–44	1731 (74.6)	46 (2.0)	544 (23.4)	
45–54	1717 (77.9)	63 (2.9)	423 (19.2)	
55–64	1512 (77.1)	73 (3.7)	377 (19.2)	
65–74	922 (75.6)	101 (8.3)	196 (16.1)	
≥ 75	457 (80.6)	51 (9.0)	59 (10.4)	
**Sex**				< 0.001
Women	4074 (91.2)	46 (1.0)	346 (7.7)	
Men	2265 (59.5)	288 (7.6)	1253 (32.9)	
**Marital status**				0.197
Non-married	53 (79.1)	5 (7.5)	9 (13.4)	
Married	6285 (76.6)	328 (4.0)	1589 (19.4)	
**Education, year**				< 0.001
Illiterate	499 (85.3)	29 (5.0)	57 (9.7)	
1–5	672 (80.0)	44 (5.2)	124 (14.8)	
6–12	3203 (74.5)	170 (4.0)	927 (21.6)	
> 12	1961 (77.2)	90 (3.5)	490 (19.3)	
**Body mass index, kg/m**^**2**^				< 0.001
< 20	148 (65.2)	8 (3.5)	71 (31.3)	
20–24.9	1532 (73.8)	85 (4.1)	458 (22.1)	
25–29.9	2608 (76.2)	146 (4.3)	670 (19.6)	
30–34.9	1434 (79.8)	66 (3.7)	296 (16.5)	
≥ 35	564 (82.7)	24 (3.5)	94 (13.8)	
**Physical activity**				< 0.001
Low	1135 (78.3)	77 (5.3)	237 (16.4)	
Intermediate	3712 (78.0)	176 (3.7)	874 (18.4)	
High	1459 (73.0)	80 (4.0)	460 (23.0)	
**Opium consumption**				< 0.001
No	6245 (80.0)	274 (3.5)	1287 (16.5)	
Yes	89 (20.2)	58 (13.2)	293 (66.6)	
**Alcohol consumption**				< 0.001
No	6094 (81.3)	247 (3.3)	1158 (15.4)	
Yes	231 (31.2)	84 (11.3)	426 (57.5)	
**Hypertension**				< 0.001
No	4478 (75.3)	191 (3.2)	1279 (21.5)	
Yes	1860 (80.1)	143 (6.2)	319 (13.7)	
**Diabetes mellitus**				< 0.001
No	5319 (76.2)	256 (3.7)	1401 (20.1)	
Yes	1020 (78.8)	78 (6.0)	197 (15.2)	
**Dyslipidemia**				< 0.001
No	4211 (75.6)	190 (3.4)	1171 (21)	
Yes	2127 (78.8)	144 (5.3)	427 (15.8)	
**Chronic kidney disease**				< 0.001
No	6287 (76.7)	323 (3.9)	1591 (19.4)	
Yes	52 (73.2)	11 (15.5)	8 (11.3)	
**Chronic lung disease**				0.009
No	6131 (76.6)	314 (3.9)	1555 (19.4)	
Yes	202 (75.9)	20 (7.5)	44 (16.5)	
**Coronary artery disease**				< 0.001
No	5800 (77.4)	245 (3.3)	1452 (19.4)	
Yes	538 (69.5)	89 (11.5)	147 (19.0)	

Data are presented as mean ± standard deviation for continuous and number (Percentage calculated for rows) for categorical variables

^*^P < 0.05 was considered significant

### Current tobacco users

Current tobacco users had a mean age of 51.7 ± 12.02 years, and the prevalence of current tobacco use showed a downward trend with advancing age (35–44 years: 23.4% vs. ≥ 75 years: 10.4%; *P* < 0.001). In addition, a substantially higher proportion of men were current tobacco users compared to women (32.9% vs. 7.7%, respectively; *P* < 0.001). Current tobacco use was higher in those with more years of education (> 12 years: 19.3% vs. illiterate: 9.7%, *P* < 0.001), higher physical activity (high: 23.0% vs. low: 16.4%, *P* < 0.001), lower BMI levels (< 20 kg/m^2^: 31.3% vs. ≥ 35 kg/m^2^: 13.8%, *P* < 0.001), alcohol drinkers (drinkers: 57.5% vs. non-drinkers: 15.4%, *P* < 0.001), and opium users (users: 66.6% vs. non-users: 16.5%, *P* < 0.001). In addition, those with preexisting comorbidities, including hypertension, diabetes mellitus, dyslipidemia, chronic kidney disease, chronic lung disease, and CAD, had a lower prevalence of current tobacco use compared to those without these conditions (Table 1).

### Former tobacco users

In our study population, 334 (4.0%) participants were former tobacco users, resulting in an age- and sex-weighted former tobacco use prevalence of 3.7% (95% CI: 2.9 – 4.7) in Tehran. Former tobacco users were significantly older than current tobacco users (61.0 vs. 51.7 years, *P* < 0.001), and their prevalence significantly increased with advancing age (35–44 years: 2.0% vs. ≥ 75 years: 9.0%, *P* < 0.001). Similar to current tobacco users, a higher percentage of men were former tobacco users than women (7.6% vs. 1.0%, *P* < 0.001). The proportion of former tobacco users was fairly similar across different levels of education (> 12 years: 3.5% vs. illiterate: 5.0%, *P* < 0.001), physical activity (high: 4.0% vs. low: 5.3%, *P* < 0.001), and BMI categories (< 20 kg/m^2^: 3.5% vs. ≥ 35 kg/m^2^: 3.5%, *P* < 0.001). However, there was an upward trend in the prevalence of former tobacco users among alcohol drinkers (drinkers: 11.3% vs. non-drinkers: 3.3%, *P* < 0.001) and opium users (users: 13.2% vs. non-users: 3.5%, *P* < 0.001). In contrast with current tobacco users, the prevalence of former tobacco use was higher among those with preexisting hypertension (6.2% vs. 3.2%, *P* < 0.001), diabetes mellitus (6.0% vs. 3.7%, *P* < 0.001), dyslipidemia (5.3% vs. 3.4%, *P* < 0.001), chronic kidney disease (15.5% vs. 3.9%, *P* < 0.001), chronic lung disease (7.5% vs. 3.9%, *P* = 0.009), and CAD (11.5% vs. 3.3%, *P* < 0.001) compared to those lacking these conditions.

### Cigarette smoking

**Fig. 2:**
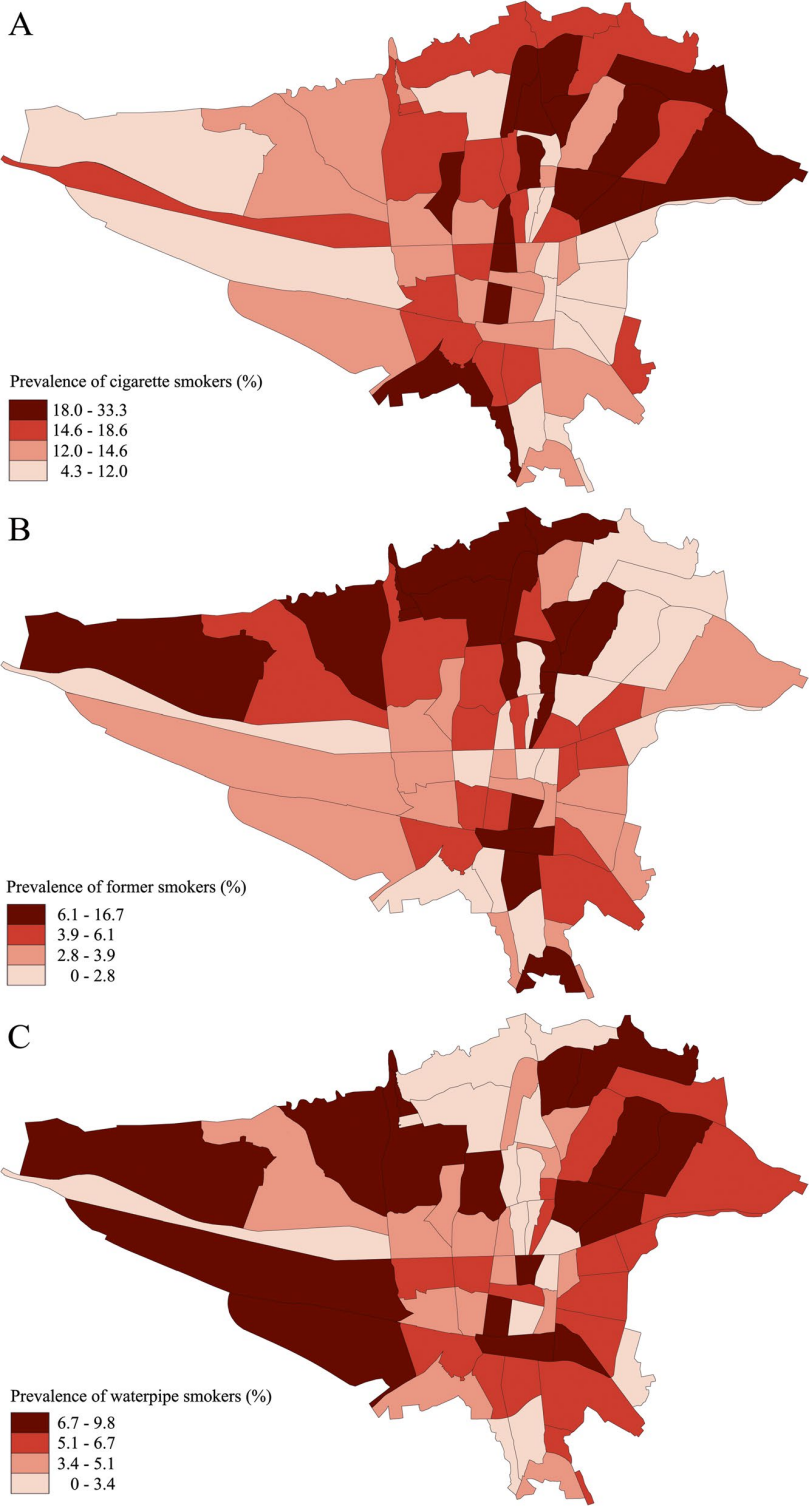
The geographic distribution of (**A**) current cigarette smokers, **B** former cigarette smokers, and (**C**) current waterpipe users in Tehran

A total of 1231 (14.9%) participants were current cigarette smokers, which constituted 77.0% of current tobacco users in our study. Hence, the age- and sex-weighted prevalence of current cigarette smoking was 14.9% (95% CI: 13.4 – 16.6) among the adult residents of Tehran. The median amount of cigarette use was 12.5 (IQR: 5–25) packs/per year. The geographical distribution of current cigarette smokers was higher in the North Eastern and some Southern districts of Tehran (Fig. 2A). The prevalence of current and former cigarette smokers within baseline characteristic subgroups is shown in Table 2.

**Table 2:** The prevalence of cigarette, waterpipe, and pipe users in the Tehran Cohort Study

	**Cigarette**		**Waterpipe**		**Pipe**	
**Characteristic**	**Former smoker** (*n* = 350)	**Current smoker** (*n* = 1231)	**Former user** (*n* = 44)	**Current user** (*n* = 449)	**Former user** (*n* = 9)	**Current user** (*n* = 44)
**Age, year**	60.6 ± 13.00	53.2 ± 11.88	54.4 ± 16.69	46.1 ± 10.91	54.2 ± 15.86	55.1 ± 12.88
**Age category, year**						
35–44	49 (2.1)	343 (14.8)	19 (0.8)	259 (11.2)	4 (0.2)	10 (0.4)
45–54	67 (3.0)	345 (15.7)	5 (0.2)	91 (4.1)	1 (0.0)	10 (0.5)
55–64	83 (4.2)	319 (16.3)	6 (0.3)	65 (3.3)	1 (0.1)	16 (0.8)
65–74	103 (8.5)	173 (14.4)	6 (0.5)	24 (2.0)	2 (0.2)	4 (0.3)
≥ 75	48 (8.5)	51 (9.1)	8 (1.4)	10 (1.8)	1 (0.2)	4 (0.7)
**Sex**						
Women	40 (0.9)	219 (4.9)	12 (0.3)	148 (3.3)	0 (0.0)	5 (0.1)
Men	310 (8.2)	1012 (26.8)	32 (0.8)	301 (7.9)	9 (0.2)	39 (1.0)
**Marital status**						
Non-married	4 (6.0)	5 (7.5)	2 (3.0)	4 (6.1)	0 (0.0)	0 (0.0)
Married	346 (4.2)	1225 (15.0)	41 (0.5)	445 (5.4)	9 (0.1)	44 (0.5)
**Education, year**						
Illiterate	26 (4.5)	47 (8.1)	6 (1.0)	11 (1.9)	0 (0.0)	1 (0.2)
1–5	42 (5.0)	100 (12.0)	6 (0.7)	29 (3.5)	0 (0.0)	1 (0.1)
6–12	190 (4.4)	722 (16.8)	21 (0.5)	259 (6.0)	2 (0.0)	18 (0.4)
> 12	92 (3.6)	361 (14.3)	10 (0.4)	150 (5.9)	7 (0.3)	24 (0.9)
**Body mass index, kg/m**^**2**^						
< 20	8 (3.6)	67 (29.8)	0 (0.0)	5 (2.2)	0 (0.0)	1 (0.4)
20–24.9	88 (4.3)	384 (18.6)	12 (0.6)	101 (4.9)	0 (0.0)	11 (0.5)
25–29.9	151 (4.4)	500 (14.6)	20 (0.6)	202 (5.9)	7 (0.2)	21 (0.6)
30–34.9	71 (4.0)	208 (11.6)	10 (0.6)	107 (6.0)	1 (0.1)	5 (0.3)
≥ 35	27 (4.0)	64 (9.4)	2 (0.3)	33 (4.8)	1 (0.1)	5 (0.7)
**Physical activity**						
Low	77 (5.3)	206 (14.3)	11 (0.8)	44 (3.0)	2 (0.1)	7 (0.5)
Intermediate	181 (3.8)	666 (14.0)	26 (0.5)	253 (5.3)	5 (0.1)	15 (0.3)
High	91 (4.6)	336 (16.9)	6 (0.3)	146 (7.3)	2 (0.1)	19 (1.0)
**Opium consumption**						
No	283 (3.6)	946 (12.2)	35 (0.4)	414 (5.3)	4 (0.1)	35 (0.4)
Yes	65 (14.9)	269 (61.6)	9 (2.1)	30 (6.9)	5 (1.1)	9 (2.1)
**Alcohol consumption**						
No	256 (3.4)	888 (11.9)	25 (0.3)	308 (4.1)	1 (0.0)	29 (0.4)
Yes	91 (12.4)	330 (44.8)	19 (2.6)	138 (18.8)	7 (1.0)	14 (1.9)
**Hypertension**						
No	210 (3.5)	969 (16.4)	28 (0.5)	385 (6.5)	6 (0.1)	31 (0.5)
Yes	140 (6.1)	261 (11.3)	16 (0.7)	64 (2.8)	3 (0.1)	13 (0.6)
**Diabetes mellitus**						
No	272 (3.9)	1077 (15.5)	40 (0.6)	398 (5.7)	6 (0.1)	40 (0.6)
Yes	78 (6.1)	153 (11.9)	4 (0.3)	51 (4.0)	3 (0.2)	4 (0.3)
**Dyslipidemia**						
No	206 (3.7)	889 (16.0)	31 (0.6)	347 (6.2)	4 (0.1)	28 (0.5)
Yes	144 (5.4)	341 (12.7)	13 (0.5)	102 (3.8)	5 (0.2)	16 (0.6)
**Chronic kidney disease**						
No	339 (4.2)	1224 (15.0)	44 (0.5)	446 (5.5)	9 (0.1)	44 (0.5)
Yes	11 (15.5)	7 (9.9)	0 (0.0)	3 (4.2)	0 (0.0)	0 (0.0)
**Chronic lung disease**						
No	330 (4.1)	1195 (15)	40 (0.5)	438 (5.5)	8 (0.1)	43 (0.5)
Yes	20 (7.5)	36 (13.6)	4 (1.5)	11 (4.1)	1 (0.4)	1 (0.4)
**Coronary artery disease**						
No	258 (3.5)	1103 (14.8)	39 (0.5)	433 (5.8)	8 (0.1)	34 (0.5)
Yes	92 (12.0)	128 (16.7)	5 (0.7)	16 (2.1)	1 (0.1)	10 (1.3)

Data are presented as mean ± standard deviation for continuous and number (percentage calculated for rows) for categorical variables

### Current cigarette smokers

**Fig. 3:**
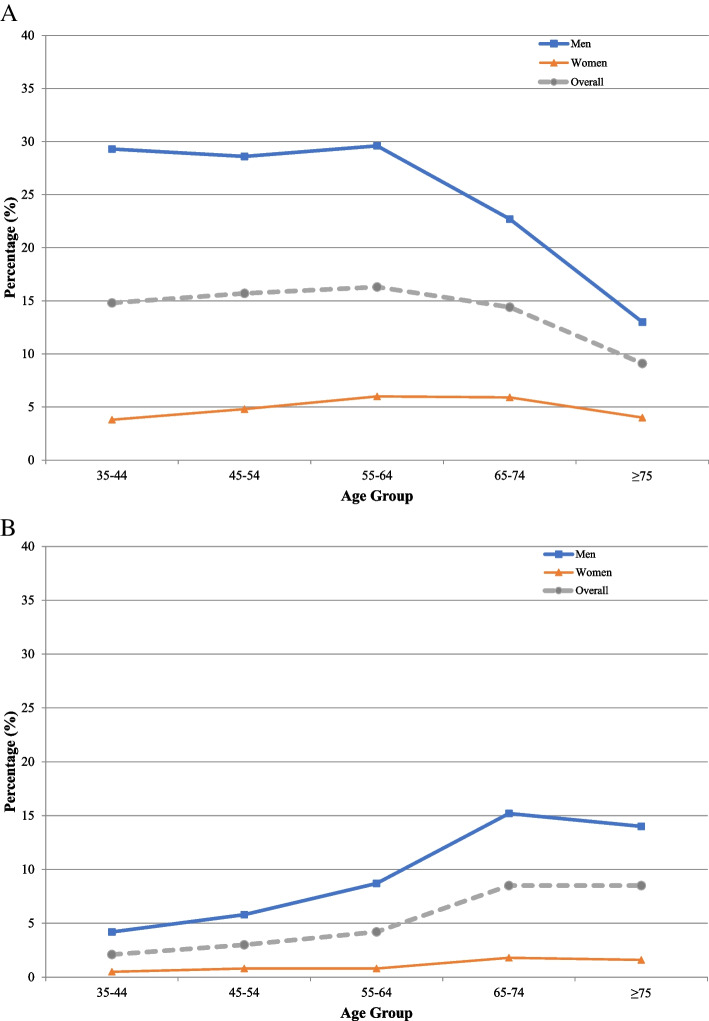
Percentage of participants with (**A**) current and (**B**) former cigarette smoking based on age and sex

The mean age of current cigarette smokers was 53.2 ± 11.88 years, and the prevalence of current cigarette smoking had a steady trend with advancing age, reaching a peak of 16.3% in those aged 55–64 years (Fig. 3A, Supplementary Table 2). The prevalence of cigarette smoking was substantially higher in men than women (26.8% vs. 4.9%), with a male/female ratio of 5.47. Moreover, current cigarette smoking was higher among participants with more years of education (> 12 years: 14.3% vs. illiterate: 8.1%), lower BMI levels (< 20 kg/m^2^: 29.8% vs. ≥ 35 kg/m^2^: 9.4%), and higher physical activity (high: 16.9% vs. low: 14.3%), alcohol drinkers (drinkers: 44.8% vs. non-drinkers: 11.9%), and opium users (users: 61.6% vs. 12.2%). Furthermore, participants with preexisting hypertension, diabetes mellitus, dyslipidemia, chronic kidney disease, chronic lung disease, and CAD had lower proportions of current cigarette smokers than those without these comorbidities (Table 2).

### Former cigarette smokers

A total of 350 (4.2%) participants were former cigarette smokers in our study population. Former cigarette smokers were estimated to have an age- and sex-weighted prevalence of 3.9% (95% CI: 3.1 – 4.9) among the adult residents of Tehran. We observed that former cigarette smoking was highest in Tehran's Northern and North Western districts (Fig. 2B). These individuals had a mean age of 60.6 ± 13.00 years, and a greater proportion of men were former cigarette smokers than women (8.2% vs. 0.9%). Former cigarette smokers were considerably older than current smokers (60.6 vs. 53.2 years), and their frequency increased with advancing age (35–44 years: 2.1% vs. ≥ 75 years: 8.5%) (Fig. 3B, Supplementary Table 2). There was no difference in the prevalence of former cigarette smokers within different levels of education, BMI, and physical activity (Table 2). However, the prevalence of former cigarette smoking was higher among alcohol drinkers (drinkers: 12.4% vs. non-drinkers: 3.4%) and opium users (14.9% vs. non-users: 3.6%). In contrast with current cigarette smokers, a higher proportion of individuals with preexisting hypertension (6.1% vs. 3.5%), diabetes mellitus (6.1% vs. 3.9%), dyslipidemia (5.4% vs. 3.7%), chronic kidney disease (15.5% vs. 4.2%), chronic lung disease (7.5% vs. 4.1%), and CAD (12.0% vs. 3.5%) were former cigarette smokers compared to those without these conditions.

### Waterpipe and pipe use

A total of 449 (5.4%) participants were current waterpipe users, and 44 (0.5%) participants were current pipe users in our study. This translates to 28.1% and 2.8% of current tobacco users in our study being waterpipe and pipe users, respectively. The age- and sex-weighted prevalence of current waterpipe and pipe users in Tehran was 6.1% (95% CI: 3.1 – 7.1) and 0.52% (95% CI: 0.27 – 0.94), respectively. As regards the geographic distribution, the Northern districts of Tehran had the least prevalence of waterpipe use (Fig. 2C). The prevalence of current and former waterpipe and pipe users within baseline characteristic subgroups is shown in Table 2.

### Current waterpipe use

**Fig. 4:**
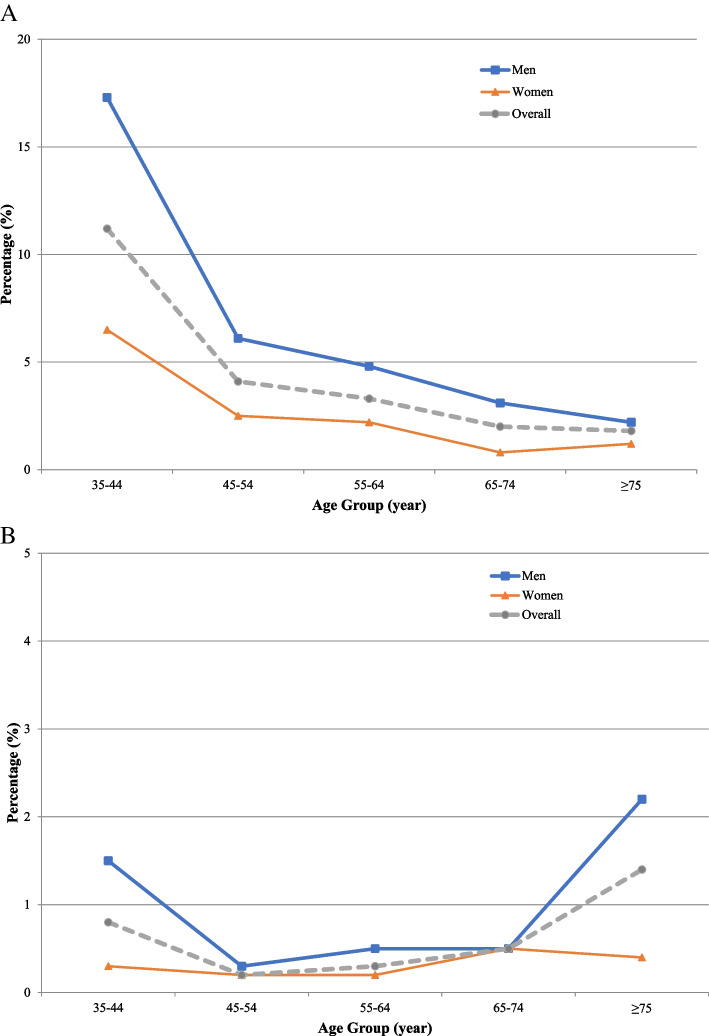
Percentage of participants with (**A**) current and (**B**) former waterpipe use based on age and sex

Current waterpipe users had a mean age of 46.1 ± 10.91 years. The prevalence of current waterpipe use was substantially higher among younger individuals and showed a declining trend with advancing age (35–44 years: 11.2% vs. ≥ 75 years: 1.8%) (Fig. 4A, Supplementary Table 2). A greater proportion of men reported current waterpipe use compared to women (7.9% vs. 3.3%), and our study's male/female ratio for waterpipe users was 2.39. In addition, the prevalence of current waterpipe use was higher among those with more years of education (> 12 years: 5.9% vs. illiterate: 1.9%), higher BMI levels (≥ 35 kg/m^2^: 4.8% vs. < 20 kg/m^2^: 2.2%), and higher physical activity (high: 7.3% vs. low: 3.0%), alcohol drinkers (drinkers: 18.8% vs. non-drinkers: 4.1%), and opium users (users: 6.9% vs. non-users: 5.3%). Furthermore, the proportion of waterpipe users was relatively lower in participants with preexisting hypertension (2.8% vs. 6.5%), diabetes mellitus (4.0% vs. 5.7%), dyslipidemia (3.8% vs. 6.2%), chronic kidney disease (4.2% vs. 5.5%), chronic lung disease (4.1% vs. 5.1%), and CAD (2.1% vs. 5.1%) compared to those without these preexisting conditions.

### Former waterpipe use

A total of 44 participants were former waterpipe users in our study. These individuals had a considerably higher mean age than current waterpipe users (54.4 ± 16.69 vs. 46.1 ± 10.91 years), and the proportion of former waterpipe users was slightly higher in men compared to women (0.8% vs. 0.3%) (Fig. 4B, Supplementary Table 2). There was no difference in the proportion of former waterpipe users with different levels of education, BMI, physical activity, alcohol drinkers, opium users, and preexisting comorbidities (Table 2).

### Pipe users

Participants with current pipe use had a mean age of 55.1 ± 12.88 years, which was similar to those with former pipe use (Table 2). The age distribution of current pipe users was relatively steady among all age groups (35–44 years: 0.4% vs. ≥ 75 years: 0.7%). However, the proportion of current pipe users was substantially higher in men than in women (1.0% vs. 0.1%). Due to the small number of participants with current and former pipe use, evaluation of the within-subgroup prevalence of current or former pipe users was underpowered.

### Determinants of tobacco use

Lastly, the adjusted model emerged that age was significantly associated with tobacco use, with older individuals being less likely to use tobacco (Table 3). Specifically, the odds of tobacco use decreased by 24.8%, 21.6%, 42.6%, and 64.4% among individuals aged 45–54, 55–64, 65–74, and ≥ 75 years, respectively, compared to those aged 35–44 years. Additionally, men had 4.3 times higher odds of tobacco use than women (OR: 4.316, *P* < 0.001). Interestingly, opium (OR: 5.557, *P* < 0.001) and alcohol consumption (OR: 4.737, *P* < 0.001) were strongly associated with tobacco use. Besides, participants with higher BMI have lower odds of using tobacco, with statistically significant differences for the 20–24.9 (OR: 0.690, *P* = 0.04), 25–29.9 (OR: 0.584, *P* = 0.002), and 30–34.9 (OR: 0.603, *P* = 0.007) BMI categories. Education was not significantly associated with tobacco use although there was a trend twaord those who had a 6–12 years of education. On the other hand, hypertension was negatively associated with tobacco use, with users having 22.6% lower odds than non-users (OR: 0.774, *P* = 0.005). However, physical activity, diabetes mellitus, dyslipidemia, chronic kidney disease, chronic lung disease, and coronary artery disease were not statistically associated with tobacco use.

**Table 3:** Multivariable analysis of risk factors associated with tobacco use among Tehran cohort study participants

Characteristic^a^	Adjusted OR (95%CI)	*P*-value^#^
Age category, year		
35–44	Ref	
45–54	0.752 (0.636–0.888)	0.001
55–64	0.784 (0.653–0.942)	0.009
65–74	0.574 (0.456–0.722)	< 0.001
≥ 75	0.356 (0.250–0.507)	< 0.001
Male sex	4.316 (3.737–4.984)	< 0.001
Being married	1.949 (0.806–4.712)	0.138
Education, year		
Illiterate	Ref	< 0.001
1–5	1.158 (0.784–1.710)	0.461
6–12	1.389 (0.988–1.954)	0.059
> 12	0.980 (0.687–1.398)	0.913
Body mass index, kg/m2		
< 20	Ref	0.014
20–24.9	0.690 (0.484–0.983)	0.04
25–29.9	0.584 (0.412–0.827)	0.002
30–34.9	0.603 (0.419–0.869)	0.007
≥ 35	0.682 (0.447–1.039)	0.075
Physical activity		
Low	Ref	0.738
Intermediate	1.077 (0.891–1.302)	0.444
High	1.075 (0.870–1.329)	0.504
Opium consumption	5.557 (4.390–7.035)	< 0.001
Alcohol consumption	4.737 (3.955–5.673)	< 0.001
Hypertension	0.774 (0.649–0.925)	0.005
Diabetes mellitus	0.903 (0.737–1.106)	0.323
Dyslipidemia	0.903 (0.773–1.055)	0.199
Chronic kidney disease	0.801 (0.342 -1.877)	0.61
Chronic lung disease	0.867 (0.588–1.278)	0.472
Coronary artery disease	0.896 (0.701–1.145)	0.382

*CI* Confidence interval, *OR* Odds ratio

^#^ *P* < 0.05 was considered significant

^a^All variables are included in the multivariable logistic regression model for calculating the adjusted odds ratios

## Discussion

This study used data from a large sample of adult residents of Tehran participating in TeCS. The prevalence of current tobacco use, cigarette smoking, waterpipe, and pipe use in Tehran was 19.8%, 14.9%, 6.1%, and 0.5%, respectively. Former tobacco users constituted 3.7% of adult residents of Tehran. Tobacco use was widespread in younger participants, men, those with higher levels of education, lower BMI, higher physical activity, and opium and alcohol users. Waterpipe users were considerably younger and had a lower male/female ratio than cigarette smokers. Moreover, our findings highlight the importance of addressing opium and alcohol consumption as risk factors for tobacco use and suggest the need for targeted interventions to reduce tobacco use among men and younger individuals.

The 2016 STEPwise approach for the surveillance of risk factors study showed that 14.1% of the Iranian population over 18 years old were current tobacco users [10]. Regarding the mentioned study, the prevalence of tobacco smoking in Tehran province was reported to be 17.1%. In addition, the Tehran Lipid and Glucose Study (TLGS) disclosed that the prevalence of tobacco smoking in adults over 20 years increased from 25.5% to 35.4% among men and 3.4% to 6.8% among women from 1999 to 2011 [15]. Moreover, the Golestan Cohort Study (GCS), which included 50 045 participants (aged 40–75) from northeastern Iran, indicated that 17% of the participants reported a history of smoking tobacco [16]. As highlighted by a related study, Iranians' tobacco smoking prevalence remained unchanged until 2016 [17]. Based on the current study, we estimate that one in five adults were current tobacco users. It is essential to note that our study population included only adults aged 35 years and older. Moreover, among Iranian adolescents, the prevalence of tobacco smoking was estimated at 9% [18]. Therefore, it is important to consider that young adults and adolescents also use tobacco products, affecting the overall estimation of tobacco use in the population.

Prior investigations have proposed an age-dependent trend in the prevalence of tobacco users [2, 19]. Consistently, our results showed that the overall prevalence of tobacco use was higher in younger age groups and declined with advancing age. However, this downward trend was not similar across different products of tobacco. While this decreasing trend was evident among current waterpipe users, the high rate of current cigarette smoking remained relatively similar among those aged 35–75 years. Waterpipe users were almost seven years younger than cigarette smokers and pipe users. These findings indicate that while targeting younger individuals might be reasonable in preventing waterpipe use, control programs should be implemented for a broader age spectrum to tackle cigarette smoking sufficiently. Waterpipe use is also spreading among the youth [8, 20]. This might be partially explained by producing flavored tobacco products over the past years. Evidence suggests that one session of waterpipe use leads to more nicotine exposure, particulate matter, and carbon monoxide than cigarette smoking [21–23]. Therefore, policymakers should emphasize educating adolescents and younger adults about waterpipe use's harms and adverse effects.

Gender differences in tobacco smoking habits are of great importance in health policies. The global prevalence of tobacco use is generally higher in men than women; however, there is a large variability between the two sexes across different geographical regions [24]. Epidemiological data from European and North American countries show a slight difference in smoking prevalence between men and women [25, 26]. On the contrary, there is a substantial difference in the proportion of tobacco users between the two sexes in the Middle Eastern populations, and the prevalence of tobacco use in Middle Eastern women is considerably lower than in the Western regions [25–28]. Consistent with these findings, the prevalence of tobacco use was approximately four-fold in men compared to women in Tehran. Although cigarettes were the most common product of tobacco smoked among both sexes, the gap between men and women was more pronounced among cigarette smokers than waterpipe users. While the proportion of cigarette smokers was almost five times higher in men than women, the prevalence of waterpipe use was twice as much in men compared to women. We hypothesize that these disparities in smoking behavior between the two sexes in Tehran possibly stem from cultural backgrounds that consider cigarette smoking shameful in women. Additionally, this issue was exacerbated by a lack of awareness about waterpipe's harmful effects, social acceptance of waterpipe usage over cigarettes, the traditional role of waterpipes as a recreational tool in Iranian society, and inadequate laws limiting the use of waterpipes in public places [29]. Previous literature depicted that women, especially those with a university education and younger age group (15–24 years), were at the most risk of waterpipe use [30]. Moreover, quitting may seem more difficult due to social acceptability than cigarette smoking in this group [31]. Overall, the significant gender gap among cigarette and waterpipe users in Tehran should guide the development of more efficient smoking cessation strategies.

Multiple studies have shown that lower education is associated with a higher risk of tobacco use [32–35]. However, adult residents of Tehran with a higher education level were more frequently tobacco users, and those with secondary level education had the highest prevalence of cigarette smoking and waterpipe use. Since a similar pattern was also observed in other local cross-sectional studies, future studies are demanding to explore this pattern further and the determinants of tobacco use among the well-educated Iranian population [36, 37]. Additionally, the proportion of tobacco users was higher among those with lower BMI and higher physical activity levels. Moreover, tobacco use was substantially higher among opium and alcohol users. This is supported by a previous study suggesting a four-fold increase in the risk of cigarette smoking among opium users [38]. These findings accentuate the need for a multi-disciplinary approach to controlling tobacco use, targeting different behavioral risk factors.

Former tobacco users were approximately ten years older than current users. The proportion of former tobacco smokers increased with advancing age, and a greater percentage of men reported a history of previous tobacco use than women. As expected, former cigarette smoking and waterpipe use was higher in participants with preexisting comorbidities. These findings suggest that developing chronic comorbidities later in life (e.g., hypertension, diabetes mellitus, dyslipidemia, chronic lung disease, etc.) might be the main factor that forces individuals to quit smoking. Subsequently, previous evidence illustrated that diabetes mellitus was significantly associated with former smoking status, while hypertension and dyslipidemia diagnoses did not correlate substantially with quitting smoking [39]. Also, many adults with documented CAD were current cigarette smokers compared to those without CAD. These findings accentuate the importance of implementing the MPOWER measures the World Health Organization recommended to facilitate smoking cessation, especially among those with comorbidities [40].

As opposed to previous studies, we found that older adults are less likely to use tobacco [10, 41]. However male gender was independent determinant for tobacco use, aligning with prior studies [41, 42]. Additionally, our findings indicated a negative association between hypertension and tobacco use (*P* = 0.005). This finding may seem counterintuitive but could be explained by confounding factors or other underlying mechanisms. However, further research may be needed to clarify this relationship in our study population.

### Limitations

The main strength of this study is that we provided comprehensive data on tobacco smoking in Tehran for the first time, and our results can be a cornerstone for future studies and public health plans. However, it also has some limitations. Like every cross-sectional study, non-responders are a barrier to reporting the actual prevalence of smoking in Tehran. Since we did not have any data from the non-responders, it is not possible to report how this group differed from the participants. Due to the multi-purpose nature of TeCS objectives, we only asked general questions about tobacco use status from participating individuals. This was due to time restrictions for data collection as patients had to be interviewed for multiple questionnaires and anthropometric measurements were taken in a half-day session. Therefore, we lack some detailed data on smoking behaviors, such as the age of beginning to smoke, the number of quit attempts, and quit strategies. Furthermore, TeCS did not include adults aged < 35 years as it was designed to track the incidence of cardiovascular diseases and the related risk factors in adults. This might have affected our estimations, especially for waterpipe use which is believed to be more popular among the youth.

## Conclusion

The prevalence of tobacco use among the adult residents of Tehran, a heavily-populated metropolis in the Middle East, is concerning. Although cigarette smokers mainly drove this high prevalence, waterpipe use also accounted for approximately one-third of tobacco users. Compared to Western regions, the observed differences in smoking behavior and epidemiology of cigarette smokers and waterpipe users in Tehran warrant exclusively tailored control programs for smoking cessation. These programs should specifically consider the large gender gaps observed in the prevalence of cigarette and waterpipe users in Middle Eastern populations.

## Supplementary Information

**Additional file 1: Supplementary Table S1. **Baseline characteristics of the Tehran Cohort Study participants. **Supplementary Table S2. **Prevalence of current and former cigarette, waterpipe, and pipe use in the Tehran Cohort study, stratified by age and sex.

## Data Availability

The data underlying this article will be shared at a reasonable request to the corresponding author.

## References

[1] Collaborators GBDRF (2016). Global, regional, and national comparative risk assessment of 79 behavioural, environmental and occupational, and metabolic risks or clusters of risks, 1990–2015: a systematic analysis for the Global Burden of Disease Study 2015. Lancet.

[2] Collaborators GBDT (2017). Smoking prevalence and attributable disease burden in 195 countries and territories, 1990–2015: a systematic analysis from the Global Burden of Disease Study 2015. Lancet.

[3] WHO global report on trends in prevalence of tobacco smoking 2000–2025, third edition. Geneva: World Health Organization; 2019.

[4] WHO report on the global tobacco epidemic (2021). addressing new and emerging products.

[5] Maziak W, Taleb ZB, Bahelah R, Islam F, Jaber R, Auf R, Salloum RG (2015). The global epidemiology of waterpipe smoking. Tob Control.

[6] El-Zaatari ZM, Chami HA, Zaatari GS (2015). Health effects associated with waterpipe smoking. Tob Control.

[7] Montazeri Z, Nyiraneza C, El-Katerji H, Little J (2017). Waterpipe smoking and cancer: systematic review and meta-analysis. Tob Control.

[8] Bhatnagar A, Maziak W, Eissenberg T, Ward KD, Thurston G, King BA, Sutfin EL, Cobb CO, Griffiths M, Goldstein LB (2019). Water Pipe (Hookah) Smoking and Cardiovascular Disease Risk: A Scientific Statement From the American Heart Association. Circulation.

[9] Dai X, Gakidou E, Lopez AD (2022). Evolution of the global smoking epidemic over the past half century: strengthening the evidence base for policy action. Tob Control.

[10] Varmaghani M, Sharifi F, Mehdipour P, Sheidaei A, Djalalinia S, Gohari K, Modirian M, Pazhuheian F, Peykari N, Haghshenas R (2020). Prevalence of Smoking among Iranian Adults: Findings of the National STEPs Survey 2016. Arch Iran Med.

[11] Nemati S, Rafei A, Freedman ND, Fotouhi A, Asgary F, Zendehdel K (2017). Cigarette and Water-Pipe Use in Iran: Geographical Distribution and Time Trends among the Adult Population; A Pooled Analysis of National STEPS Surveys, 2006–2009. Arch Iran Med.

[12] Abdollahpour I, Mansournia MA, Salimi Y, Nedjat S (2019). Lifetime prevalence and correlates of smoking behavior in Iranian adults' population; a cross-sectional study. BMC Public Health.

[13] Fotouhi A, Khabazkhoob M, Hashemi H, Mohammad K (2009). The prevalence of cigarette smoking in residents of Tehran. Arch Iran Med.

[14] Shafiee A, Saadat S, Shahmansouri N, Jalali A, Alaeddini F, Haddadi M, Tajdini M, Ashraf H, Omidi N, Masoudkabir F (2021). Tehran cohort study (TeCS) on cardiovascular diseases, injury, and mental health: Design, methods, and recruitment data. Global Epidemiology.

[15] Parizadeh D, Momenan AA, Amouzegar A, Azizi F, Hadaegh F (2018). Tobacco Smoking: Findings from 20 Years of the Tehran Lipid and Glucose Study. Int J Endocrinol Metab.

[16] Etemadi A, Khademi H, Kamangar F, Freedman ND, Abnet CC, Brennan P, Malekzadeh R (2017). Hazards of cigarettes, smokeless tobacco and waterpipe in a Middle Eastern Population: a Cohort Study of 50 000 individuals from Iran. Tob Control.

[17] Sohrabi MR, Abbasi-Kangevari M, Kolahi AA (2020). Current Tobacco Smoking Prevalence Among Iranian Population: A Closer Look at the STEPS Surveys. Front Public Health.

[18] Ehsani-Chimeh E, Sajadi HS, Behzadifar M, Aghaei M, Badrizadeh A, Behzadifar M, Bragazzi NL (2020). Current and former smokers among adolescents aged 12–17 years in Iran: a systematic review and meta-analysis. BMC Public Health.

[19] Welte JW, Barnes GM (2011). Tidwell M-CO, Hoffman JH: Tobacco use, heavy use, and dependence among adolescents and young adults in the United States. Subst Use Misuse.

[20] Jawad M, Lee JT, Millett C (2016). Waterpipe Tobacco Smoking Prevalence and Correlates in 25 Eastern Mediterranean and Eastern European Countries: Cross-Sectional Analysis of the Global Youth Tobacco Survey. Nicotine Tob Res.

[21] St Helen G, Benowitz NL, Dains KM, Havel C, Peng M, Jacob P (2014). Nicotine and carcinogen exposure after water pipe smoking in hookah bars. Cancer Epidemiol Biomarkers Prev.

[22] Monn C, Kindler P, Meile A, Brandli O (2007). Ultrafine particle emissions from waterpipes. Tob Control.

[23] Daher N, Saleh R, Jaroudi E, Sheheitli H, Badr T, Sepetdjian E, Al Rashidi M, Saliba N, Shihadeh A (2010). Comparison of carcinogen, carbon monoxide, and ultrafine particle emissions from narghile waterpipe and cigarette smoking: Sidestream smoke measurements and assessment of second-hand smoke emission factors. Atmos Environ (1994)..

[24] Islami F, Stoklosa M, Drope J, Jemal A (2015). Global and Regional Patterns of Tobacco Smoking and Tobacco Control Policies. Eur Urol Focus.

[25] Dorner TE, Brath H, Kautzky-Willer A (2020). Sex-specific trends in smoking prevalence over seven years in different Austrian populations: results of a time-series cross-sectional analysis. BMJ Open.

[26] Cornelius ME, Wang TW, Jamal A, Loretan CG, Neff LJ (2020). Tobacco Product Use Among Adults - United States, 2019. MMWR Morb Mortal Wkly Rep.

[27] Alali WQ, Longenecker JC, Alwotyan R, AlKandari H, Al-Mulla F, Al Duwairi Q (2021). Prevalence of smoking in the Kuwaiti adult population in 2014: a cross-sectional study. Environ Sci Pollut Res Int.

[28] Moradi-Lakeh M, El Bcheraoui C, Tuffaha M, Daoud F, Al Saeedi M, Basulaiman M, Memish ZA, AlMazroa MA, Al Rabeeah AA, Mokdad AH (2015). Tobacco consumption in the Kingdom of Saudi Arabia, 2013: findings from a national survey. BMC Public Health.

[29] Danaei M, Jabbarinejad-Kermani A, Mohebbi E, Momeni M (2017). Waterpipe Tobacco Smoking Prevalence and Associated Factors in the Southeast of Iran. Addict Health.

[30] Baheiraei A, Mirghafourvand M, Nedjat S, Mohammadi E, Mohammad-AlizadehCharandabi S (2012). Prevalence of water pipe use and its correlates in Iranian women of reproductive age in Tehran: a population-based study. Med Princ Pract.

[31] Akl EA, Jawad M, Lam WY, Co CN, Obeid R, Irani J (2013). Motives, beliefs and attitudes towards waterpipe tobacco smoking: a systematic review. Harm Reduct J.

[32] Islam MS, Saif-Ur-Rahman KM, Bulbul MMI, Singh D (2020). Prevalence and factors associated with tobacco use among men in India: findings from a nationally representative data. Environ Health Prev Med.

[33] Lim KH, Teh CH, Pan S, Ling MY, Yusoff MFM, Ghazali SM, Kee CC, Lim KK, Chong KH, Lim HL (2018). Prevalence and factors associated with smoking among adults in Malaysia: Findings from the National Health and Morbidity Survey (NHMS) 2015. Tob Induc Dis.

[34] Gilman SE, Martin LT, Abrams DB, Kawachi I, Kubzansky L, Loucks EB, Rende R, Rudd R, Buka SL (2008). Educational attainment and cigarette smoking: a causal association?. Int J Epidemiol.

[35] Sreeramareddy CT, Harper S, Ernstsen L (2018). Educational and wealth inequalities in tobacco use among men and women in 54 low-income and middle-income countries. Tob Control.

[36] Hamzeh B, Farnia V, Moradinazar M, Pasdar Y, Shakiba E, Najafi F, Alikhani M (2020). Pattern of cigarette smoking: intensity, cessation, and age of beginning: evidence from a cohort study in West of Iran. Subst Abuse Treat Prev Policy.

[37] Shuja M, Sarrafzadegan N, Roohafza HR, Sadeghi M, Ghafari M, Mohammadian M, MohammadianHafshejani A (2017). Factors Associated with Cigarette Smoking in Central Parts of Iran. Asian Pac J Cancer Prev.

[38] Fallahzadeh MA, Salehi A, Naghshvarian M, Fallahzadeh MH, Poustchi H, Sepanlou SG, Gandomkar A, Malekzadeh R (2017). Epidemiologic Study of Opium Use in Pars Cohort Study: A Study of 9000 Adults in a Rural Southern Area of Iran. Arch Iran Med.

[39] Patel K, Schlundt D, Larson C, Wang H, Brown A, Hargreaves M (2009). Chronic illness and smoking cessation. Nicotine Tob Res.

[40] World Health Organization (WHO) (2021). WHO report on the global tobacco epidemic 2021: addressing new and emerging products.

[41] Palipudi KM, Sinha DN, Choudhury S, Zaman MM, Asma S, Andes L, Dube S (2012). Predictors of tobacco smoking and smokeless tobacco use among adults in Bangladesh. Indian J Cancer.

[42] Cham B, Scholes S, Groce NE, Mindell JS: Prevalence and Predictors of Smoking among Gambian Men: A Cross-Sectional National WHO STEP Survey. Int J Environ Res Public Health. 2019;16(23):4719. 10.3390/ijerph16234719 PMC6926921 31779281

