# Supplementary material for: Epidemiology and prevalence of tobacco use in Tehran; a report from the recruitment phase of Tehran cohort study
Source: BMC Public Health. 2023 Apr 21;23:740. doi: 10.1186/s12889-023-15629-4 (PMC10122292; doi:10.1186/s12889-023-15629-4)
Supplement: Supplementary file 1 — Additional file 1: Supplementary Table S1. Baseline characteristics of the Tehran Cohort Study participants. Supplementary Table S2. Prevalence of current and former cigarette, waterpipe, and pipe use in the Tehran Cohort study, stratified by age and sex. [file 12889_2023_15629_MOESM1_ESM.docx]

**Supplements**

**Supplementary Table S1.** Baseline characteristics of the Tehran Cohort Study participants

|  | **Total population**  (n=8272) |
| --- | --- |
| **Age, year** | 53.8 ± 12.74 |
| **Age category, year** |  |
| 35-44 | 2321 (28.1) |
| 45-54 | 2203 (26.6) |
| 55-64 | 1962 (23.7) |
| 65-74 | 1219 (14.7) |
| ≥75 | 567 (6.9) |
| **Women** | 4466 (54.0) |
| **Married** | 8202 (99.2) |
| **Education, year** |  |
| Illiterate | 585 (7.1) |
| 1-5 | 840 (10.2) |
| 6-12 | 4300 (52.0) |
| >12 | 2541 (30.7) |
| **Body mass index, kg/m^2^** |  |
| <20 | 227 (2.8) |
| 20-24.9 | 2075 (25.3) |
| 25-29.9 | 3424 (41.7) |
| 30-34.9 | 1796 (21.9) |
| ≥35 | 682 (8.3) |
| **Physical activity** |  |
| Low | 1449 (17.6) |
| Intermediate | 4762 (58.0) |
| High | 1999 (24.3) |
| **Opium consumption** | 440 (5.3) |
| **Alcohol consumption** | 741 (9.0) |
| **Hypertension** | 2322 (28.1) |
| **Diabetes mellitus** | 1295 (15.7) |
| **Dyslipidemia** | 2698 (32.6) |
| **Chronic kidney disease** | 71 (0.9) |
| **Chronic lung disease** | 266 (3.2) |
| **Coronary artery disease** | 774 (9.4) |
| Data are presented as mean±standard deviation for continuous and number (percentage in the column) for categorical variables. | |

**Supplementary Table S2.** Prevalence of current and former cigarette, waterpipe, and pipe use in the Tehran Cohort study, stratified by age and sex

|  |  | **Cigarette** | | **Waterpipe** | | **Pipe** | |
| --- | --- | --- | --- | --- | --- | --- | --- |
| **Gender** | **Age group** | **Former** | **Current** | **Former** | **Current** | **Former** | **Current** |
| **Female** | **35-44** | 7 (0.5) | 50 (3.8) | 4 (0.3) | 86 (6.5) | 0 (0) | 0 (0) |
|  | **45-54** | 9 (0.8) | 58 (4.8) | 2 (0.2) | 30 (2.5) | 0 (0) | 3 (0.3) |
|  | **55-64** | 9 (0.8) | 66 (6) | 2 (0.2) | 24 (2.2) | 0 (0) | 1 (0.1) |
|  | **65-74** | 11 (1.8) | 35 (5.9) | 3 (0.5) | 5 (0.8) | 0 (0) | 0 (0) |
|  | **≥75** | 4 (1.6) | 10 (4) | 1 (0.4) | 3 (1.2) | 0 (0) | 1 (0.4) |
| **Male** | **35-44** | 42 (4.2) | 293 (29.3) | 15 (1.5) | 173 (17.3) | 4 (0.4) | 10 (1) |
|  | **45-54** | 58 (5.8) | 287 (28.6) | 3 (0.3) | 61 (6.1) | 1 (0.1) | 7 (0.7) |
|  | **55-64** | 74 (8.7) | 253 (29.6) | 4 (0.5) | 41 (4.8) | 1 (0.1) | 15 (1.8) |
|  | **65-74** | 92 (15.2) | 138 (22.7) | 3 (0.5) | 19 (3.1) | 2 (0.3) | 4 (0.7) |
|  | **≥75** | 44 (14) | 41 (13) | 7 (2.2) | 7 (2.2) | 1 (0.3) | 3 (0.9) |
| Data are presented as numbers (percentages in the row). | | | | | | | |
